# Medical futility and its challenges: a review study

**Published:** 2016-10-20

**Authors:** Maryam Aghabarary, Nahid Dehghan Nayeri

**Affiliations:** 1 PhD Student in Nursing, Nursing and Midwifery Care Research Center, Faculty of Nursing and Midwifery, Tehran University of Medical Sciences, Tehran, Iran;; 2 Professor, Nursing and Midwifery Care Research Center, Faculty of Nursing and Midwifery, Tehran University of Medical Sciences, Tehran, Iran.

**Keywords:** *Medical futility*, *Physiologic futility*, *Qualitative futility*, *Decision-making*, *Withholding of treatments*

## Abstract

Concerns over limited medical equipment and resources, particularly in intensive care units (ICUs), have raised the issue of medical futility. Medical futility draws a contrast between physician’s authority and patients’ autonomy and it is one of the major issues of end-of-life ethical decision-making. The aim of this study was to review medical futility and its challenges.

In this systematized review study, a comprehensive search of the existing literature was performed using an internet search with broad keywords to access related articles in both Persian and English databases. Finally, 89 articles were selected and surveyed.

Medical futility is a complex, ambiguous, subjective, situation-specific, value-laden, and goal-dependent concept which is almost always surrounded by some degrees of uncertainty; hence, there is no objective and valid criterion for its determination. This concept is affected by many different factors such as physicians’ and patients’ value systems, medical goals, and sociocultural and religious context, and individuals’ emotions and personal characteristics.

It is difficult to achieve a clear consensus over the concept of medical futility; hence, it should be defined and determined at an individual level and based on the unique condition of each patient.

## Introduction

Concerns over limited medical equipment and resources, particularly in intensive care units (ICUs), have raised the issue of medical futility (1-4). Advances in medical technology, increased healthcare costs, and the aging of the population have added to the importance of medical futility in recent years, so much so that the issue of medical futility has become an increasing concern (3, 5-9).

Technological advances have enabled medical experts to prolong the lives of terminally-ill patients even when there is no hope for successful treatment of their underlying pathology. In addition to generating debates on heavy healthcare costs, such practices have increased the demand for intensive care services and ICU equipment particularly by elderly people suffering from chronic conditions (5, 8). This increase in demand for intensive care services may become greater than the supplies in the near future and cause different problems (1-3). For instance, the need for ICU beds is estimated to increase by 80–93% in the subsequent 20 years. Consequently, the impending shortage of ICU beds highlights the necessity for paying greater attention to the debates over futile treatments, particularly in ICUs (2). 

Most people believe that futile treatments should not be provided; however, there are different viewpoints about what can be defined as a futile treatment (8). Differences in people’s perceptions of futile treatment have created many challenges between patients’ family members and healthcare professionals regarding continuing or discontinuing treatments (3). Contrast between physician’s authority and patients’ autonomy is another important issue which has made clinical decision-making difficult. Some individuals believe that judgment about futility of treatments is a privilege of medicine and is more valuable than patients’ autonomy (10). However, there might be instances in which patients’ family members insist on continuing treatments, while patients are reluctant to receive them and healthcare professionals believe that they are futile (2, 8, 11). Although a physician can ethically reduce the delivery of treatments which are inappropriate or futile (3), the questions ‘Is the treatment really futile?’ and ‘Who has the right to determine futility (physician, patient, or family members)? (12) are raised.

Consequently, deciding on the futility of a certain treatment is among the most sensitive health care issues which can even result in making decisions that are unethical. The sensitivity of this issue originates from the fact that the term ‘futile treatment’ is widely used in clinical reasoning as a strong reason for avoiding treatment of a patient. Accordingly, a cause of concern here is that valuable treatments are discontinued for patients who are unable to make decisions because treatments are considered to be futile (13). Similarly, treatments with small gain may be eliminated out of their presumed futility. This may finally result in patients’ premature death. Another concern in the area of futility is that essential treatments may be labeled as futile in order to cut healthcare costs (14). Accordingly, the major futility-related concerns are: ’What is futility?’ ‘How can it be defined?’ ‘What are its attributes and instances?’ ‘What factors affect people’s perceptions of it?’ ‘Who has the authority to decide upon continuation or discontinuation of futile treatments?’ ‘What factors result in the delivery of futile treatments?’ and ‘What are the consequences of futile treatments?’ This review study aims to answer these questions. The findings of this study can enhance healthcare professionals’ understanding and knowledge regarding the nature, definitions, attributes, reasons, and consequences of the concept of medical futility.

## Method

**Figure 1:**
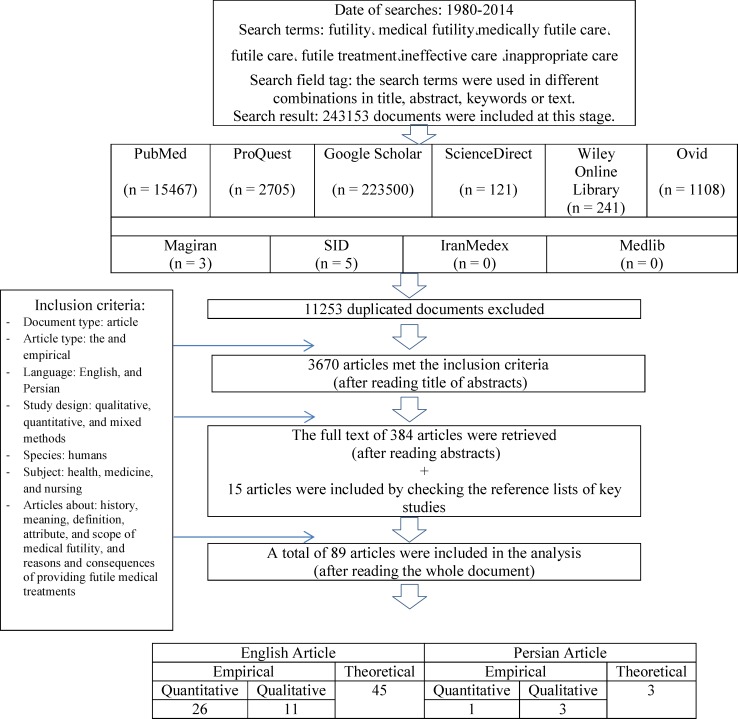
The process of searching, retrieving, and selecting the documents

This systematized review was conducted from December 2013 to April 2014. A comprehensive search was conducted via PubMed, ProQuest, Ovid, Wiley Online Library, Science Direct, and Google Scholar databases. The time interval determined in the search protocol was 1980–2014. The search keywords were futility, medical futility, medically futile care, futile care, futile treatment, ineffective care, inappropriate care, and non-beneficial care. The equivalents of these keywords in Farsi were searched in Persian databases such as Sicentific Information Database (SID), IranMedex, Magiran, and Medlib. By using these broad terms, initially, more than 10000 documents (including articles, books, and theses) were found. After excluding books, theses, duplicate articles, commentaries and letters to the editor, the titles of the articles were assessed and the irrelevant articles were excluded. The abstracts of the remaining articles were studied. Thereafter, the full text of 284 articles which met the inclusion criteria were retrieved and studied. Moreover, the reference lists of the retrieved articles were assessed. Finally, 89 articles which met the inclusion criteria were included in the final analysis. Figure 1 shows the inclusion criteria, and the process of searching, retrieving, and selecting the documents. 

## Results

***The history and emergence of the concept of medical futility***

Futility in medicine is a concept with a long history. The Hippocratic Oath includes a part which requires physicians to avoid over-treating a patient, at any cost, whose body has been swamped with diseases. Hippocrates clearly noted that medicine is unable to treat such patients (3, 15-17). Consequently, avoidance of futile treatment became an ethical obligation for physicians since the time of Hippocrates (12, 18). On the other hand, rapid advances in medical sciences and technology made it possible to manage and treat many life-threatening conditions, increased human longevity, and led to an increase in the population of elderly people. Medical technology helped physicians prolong the lives of many terminally-ill patients without having any hope for successful treatment of their underlying pathologies (8, 19). In other words, medical science reached a state in which it was able neither to prevent patients’ inevitable death nor to ignore patients whose death was imminent. Some professionals have equated such practices with prolonging the process of patients’ death, pain, and agony, and reducing their quality of life (QOL). Moreover, given the scarcity of medical equipment and the heavy burden of healthcare costs, it was considered as an ineffective, worthless, and futile practice (8, 19, 20). Therefore, the concept of medical futility was introduced in the late 1980s (19, 21, 22) in order to discontinue life-sustaining treatments for terminally-ill patients (3, 21, 23). An important question which was raised then was: ‘Does one-sided labeling of a treatment procedure as futile by a physician provide the permission for discontinuing that procedure or avoiding its administration (24)?’ The ethical challenge of such a practice was that human life cannot be decided on only by physicians, but that patients and their families also have the right to participate in the decision-making process. Therefore, the history of scientific debates about futility in medicine and medical ethics go back to the 1990s (3). 

Other experts considered the contrast between physicians’ authority and patients’ autonomy as the reason behind the emergence of this concept (25-27). The physician-patient paternalistic relationship in the past sometimes required patients to receive treatments which they did not like. Patients’ reluctance to and dissatisfaction with receiving such unwanted treatments caused the medical society to base clinical decisions and prescriptions on patient’s right to have autonomy (28). Therefore, the paternalistic physician-patient relationship was changed into a participatory relationship. However, patient’s autonomy was limited only to accepting or rejecting diagnostic and therapeutic procedures and it did not include patient’s right to ask to receive treatments (29). Thereafter, rapid advances in medical sciences created realistic and unrealistic expectations from medical technology and enhanced patient’s autonomy and authority, so much so that physicians gradually received requests from patients and their families for treatments which were considered professionally as futile, ineffective, worthless, or impossible. Consequently, some experts believe that the concept of medical futility was introduced by the medical society in order to regain its earlier paternalistic authority and position and to use it as permission for rejecting patient’s requests (16, 30). However, after some time, it was found that improper use of this concept can cause many ethical challenges.

***The lexical meaning and the definition of medical futility***

The root of the word ‘futile’ is the Latin word ‘*futtilis*’ which means worthless. The ordinary meanings of futile include ineffective, useless, unsuccessful, and meritless (9). Webster’s dictionary defines futility as ‘serving no useful purpose; completely ineffective or producing no valuable effect’ (31). The definition of this word in the Oxford English Dictionary is ‘leaky, vain, failing of the desired end through intrinsic defect’ (32).

Simply, medical futility occurs when:

1. There is a goal
2. There is an action or activity for achieving that goal
3. There is a virtual certainty that the action or the activity fails to achieve the goal

Consequently, the simplest definition of medical futility would be: ‘a clinical action which is not performed for achieving a clear goal, and hence, is not useful for the intended patient’ (15).

Many scholars considered this simple definition as inadequate, criticized it, and thus, provided different definitions for the concept and used different expressions and terms for explaining it, all of which added to the ambiguity of the concept (33). 

Table 1 shows that there are numerous definitions and terms for medical futility. Nonetheless, the most cited definition of medical futility is the definition which was provided by Schneiderman et al. (34).

**Table 1:** The definitions of the concept of medical futility in the literature

**No**	**Keyword**	**Definition**	**Author(s)**
1	Medical futility	Quantitative medical futility: “When physicians conclude (either through personal experience, experiences shared with colleagues, or consideration of published empiric data) that in the last 100 cases a medical treatment has been useless, they should regard that treatment as futile” (p.437). Qualitative medical futility: “Physicians should distinguish between an effect which is limited to some part of the patient^'^s body, and benefit which the patient has the capacity to appreciate and which improves the patient as a whole” (p.950). “If a treatment merely preserves permanent unconsciousness or cannot end dependence on intensive medical care, the treatment should be considered futile” (p.437).	Schneiderman et al. (17, 34, 35)
2	Medical futility	Medical futility “is when treatment cannot, within a reasonable probability, cure, ameliorate, improve or restore a quality of life that would be satisfactory to the patient” (p.36).	Quinn (41)
3	Medical futility	Quantitative medical futility is related to the success of a treatment in achieving its intended goals. Qualitative medical futility is related to the value of a treatment to a patient’s QOL.	Schneiderman et al. (27)
4	Medical futility Futile treatment	“An action, intervention or procedure that might be physiologically effective in a given case but cannot benefit the patient, no matter how often it is repeated. A futile treatment is not necessarily ineffective, but it is worthless either because the medical action itself is futile, (no matter what the patient s condition) or the condition of the patient makes it futile” (p.69).	Clark (38)
5	Medical futility	The concept needs to be defined individually and based on the unique condition of each patient and the desires of the patient and family members: Continuing treatments while death is certain and survival is impossible Continuing treatments while post-survival QOL is low (because of permanent physical or cognitive damage) Continuing treatments for a patient with brain death	Heland (40)
6	Medical futility	The concept needs to be defined individually and based on the unique condition of each patient: Medical futility is a state in which an intervention (either diagnostic, therapeutic, preventive, or rehabilitative) provides no benefit to the intended patient.	Aramesh (37)
7	Medical futility	Medical futility at the end of life includes the following instances: Failure to achieve goals such as saving life, prolonging life, and improving QOL Disproportionate harm-benefit ratio: imposing heavy costs or inflicting harm The concept needs to be defined individually and based on the unique condition of each patient	Jox et al. (30)
8	Medical futility	The concept needs to be defined individually and based on the unique condition of each patient: A state in which a certain intervention produces no benefit to a certain patient. The intervention may include a surgery, intravenous or oral medications, or laboratory or imaging studies.	Saettele and Kras (8)
9	Futile treatment	In the context of medicine, futile treatment is a type of care which does not fulfill the intended goals and includes: A treatment which does not provide a reasonable chance of survival A treatment which is useless or ineffective A treatment which is unsuccessful at enhancing QOL or medical utility A treatment which can never fulfill the patient’s goals The definitions 1 and 2 are the definitions of quantitative or physiologic futility and relate to alterations in the functions of organs. Perceiving and using these two definitions are associated with few problems and debates for physicians. Definitions 3 and 4 pertain to qualitative futility, are mostly holistic, and seek patient’s benefits.	Jecker et al. (36)
10	Futility/Futile care	Treatment is medically futile or non-beneficial because it offers no reasonable hope of recovery or improvement, or because the patient is permanently unable to experience any benefit.	Jones and Hunter (39)
11	Futile treatment	“Treatments that offer no physiological benefits to the patient are futile” (p.888).	Danis et al. (24)
12	Futility/Futile care	Futility is a complex concept which relates to achieving and fulfilling the intended goals. An action is considered futile once it cannot achieve its intended goals or its success is empirically improbable. Futile care is a state in which providing life-sustaining treatments produces no medical benefit for the intended patient, cannot terminate patient’s dependence on intensive medical treatments, and results in an unacceptable level of QOL.	Meltzer and Huckabay (11)
13	Futile care	“Medically futile care to mean the use of considerable resources without a reasonable hope that the patient would recover to a state of relative independence or be interactive with his or her environment” (p.1201).	Sibbald et al. (2)
14	Futile care	Futile care “consists giving clinical cares irrelevant to a nurse’s job and giving cares through which the return of patient would be impossible both physiologically and qualitatively” (p.301).	Bahramnezhad et al. (54)
15	Futile care	Futile care “is useless, ineffective care giving with wastage of resources and torment of both patients and nurses having nursing and medical aspects” (p. 235).	Yekefallah et al. (55)

 In their definition, they highlighted the difference between effect and utility in that effect is limited to a certain part of a patient’s body while utility or benefit encompasses all the aspects of a patient as a whole. According to them, a treatment which has an effect, but has no utility for a patient is considered as futile (17, 34, 35). Based on the difference between effect and utility, futility can be classified as physiologic, quantitative and normative, or qualitative futility (Table 2).

**Table 2:** The types and the examples of medical futility

**Medical futility**	**Definition**	**Examples**
Strict physiologic futility (Focuses on achieving the physiological effects of treatments.)	Treatments do not produce the intended physiological effect Treatments do not help achieve the intended physiological goals	∆ Ineffectiveness of an antibiotic against viral infection ∆ Ineffectiveness of aspirin in managing cancer ∆ The treatment is not effective in reversing a physiologic deterioration which will finally cause death. The medical diagnosis shows an inevitable death and the treatment will have no useful physiologic effect. For instance, ineffectiveness of defibrillation on asystole or conventional cardiopulmonary resuscitation for a patient with myocardial rupture.
Quantitative futility (Focuses on the success rate of a treatment.)	The chance of producing the desired effects is low or poor (less than 1%).	∆ The low success rate of saving the life of an elderly patient who suffers from end-stage hepatic cirrhosis and severe organ failure
Qualitative futility (Focuses on the value of treatments in terms of QOL.)	Treatments which have the desired physiological effects, but the effects are useless or worthless to the intended patient The effect is producible, but there are value-laden controversies on its justifiability Given the disproportionate harm-benefit ratio, the treatment has no value to patient’s QOL.	∆ A successful resuscitation which finally results in a vegetative state for the patient ∆ Poor QOL after a successful resuscitation on a patient with end-stage cancer whose survival had been estimated to be 0%–10% ∆ Prolonging survival for only two months by using costly and potentially harmful chemotherapy agents ∆ Sustaining the life of a terminally-ill patient using life-sustaining treatments (such as ventilator and vasopressors)

A brief review of the existing definitions of the concept of medical futility (Tables 1, 2, and 3) reveals that these definitions have been based on the following six foundations:

1. The probability of achieving the physiological effects which have been supposed for a medical treatment (only physicians can determine it) (12, 17, 24, 34)
2. The probability of achieving the defined goals of a treatment (physicians, patients, and family members can have roles in determining it) (11, 23, 30, 36)
3. The amount of benefit and utility which the intended treatment has for the intended patient (this is completely individual and is affected by values) (8, 11, 36-39)
4. The survival rate of the intended treatment (30, 36, 40)
5. Post-treatment quality of life (QOL) (8, 11, 17, 30, 34, 36, 40, 41)
6. The cost-effectiveness of the treatment (2, 8, 30)

The abovementioned data reveals that the probability of the success of a treatment and the value of the treatment in terms of QOL are two main themes which can be extracted from the existing definitions. However, the diversity of perceptions of acceptable probability of success and acceptable QOL has made it difficult to provide a clear and comprehensive definition of the concept (42). The main problem occurs when we decide to determine an objective border beyond which medical treatments can be considered as futile. In other words, how much should the probability of success for a treatment or QOL be in order to consider the treatment futile (12)? Most importantly, who has the authority or the competence to define and establish such borders? We could not find any clear answer to these questions in the literature; however, in the majority, it was indicated that judgment on futility is an individual concept and based on the unique conditions of each patient (8, 30, 37, 40).

***The main components in the definitions of the concept of medical futility***

The data presented in tables 1, 2, and 3 reveal that the main components of medical futility debates are goal, effect, utility, and value. 

*Goals of medicine* **:** The most fundamental component of medical futility is the goal of medicine. Determining whether a treatment is ineffective, useless, or worthless necessitates weighing it against the intended goals (11, 12, 15, 23, 30). In other words, we can talk about the effect, utility, or value of a certain treatment only when we know the goals of that treatment. In the next step, the probability of achieving the goals and the effect, utility, and value of achieving the goals are assessed (23, 42). Consequently, improbability or low probability of achieving the intended goals is among the most essential characteristics of the concept of medical futility (17, 34). The goals may include

- Successful treatment, complete recovery, returning to normal life, and gaining autonomy and the ability to interact with the surrounding environment;
- Achieving the physiological outcomes of the treatments irrespective of the quality of their effects (for instance, successful removal of excess fluids and waste products by a dialysis machine irrespective of the effect of dialysis on the survival of a dying patient);
- Saving life and preventing death;
- Improving survival and prolonging life (without inflicting pain or agony and not at any cost);
- Alleviating pain and other physical symptoms and providing comfort;
- Psychological palliation (giving hope, sympathizing, and bringing satisfaction to patients);
- Improving QOL through alleviating physical and psychological symptoms;
- Preparing the patient for a peaceful death.

The goals may change during the course of the disease and in line with the patient’s condition, medical treatments, access to equipment and facilities, and etcetera. Any change in the goals may be associated with changes in individuals’ perceptions of the utility and their judgment about the futility of a certain treatment (23). 

*Effect* **:** Effect is the result of achieving the physiological goals which have been set for a treatment while utility or outcome implies the quality of the effect. A futile treatment may exert significant effects on patients’ physiology or anatomy; however, the important point here is that the effects are not useful to the patient. Therefore, ‘utility’ is a key term in medical futility debates (37). 

*Utility* **:** Utility can be objective or subjective and physical or psychological (13). Although the meaning of utility in the area of medical futility is the direct and indirect benefits of treatments for patients, decision upon the futility or non-futility of a certain treatment is sometimes made based on the benefits of that treatment for other people (such as family members or other patients). The most prominent example in this area is hospitalizing and caring for a patient with brain death in the ICU. Given the current inabilities of medical sciences, providing life-sustaining medical treatments to such a patient is among the clearest instances of medical futility (10, 18, 40). The reason is that none of the abovementioned goals for the patient are achievable, and thus, continuing life-sustaining treatments is completely useless to the patient. On the other hand, such treatments are not futile if they are provided for the purpose of organ donation to other patients or in order to help the patient’s family members cope with and accept their patient’s death. The reason is that such practices can be beneficial to other people (including family members and other patients). 

*Value* **:** For assessing the value of a treatment, not only the probability of achieving the goals, but also the amount of benefit should be taken into account. The benefit can be measured using the benefit-harm ratio (23, 30). In other words, if achieving the intended goals inflicts heavy costs, undue pain, agony, or damage, the value of the benefit resulted from treatments is dubious. Of course, judgments about value should also be made individually and based on patients’ and their family members’ values and preferences (2, 22, 23, 40). For instance, prolonging the survival of a patient with end-stage ovarian cancer for only two months by administrating costly and potentially harmful chemotherapy agents may be considered futile and worthless by many physicians, nurses, hospital managers, and insurance companies. They may not consider a two-month increase in survival as an optimum goal and may also not consider the benefits of achieving the goal proportional to the harms of the treatment. On the other hand, the patient, who is waiting for the birth of her first grandchild in the next two months, may consider such short-term increase in survival as a desirable goal which is worth achieving. 

***Factors affecting perceptions of medical futility***

Factors which can affect individuals’ perceptions of the concept of medical futility are the conditions of patient/disease, medical goals (therapeutic or palliative), and the value system of patients, family members, and healthcare professionals. These factors are discussed in what follows.

*The conditions of patient/disease*: Patient-related and disease-related factors can contribute to the perceptions of futility or non-futility of medical treatments (Table 4). 

**Table 3:** Comparing quantitative and qualitative futility

**Quantitative futility**	**Qualitative futility**
∆ Physiologic futility ∆ Goal futility ∆ Value free	∆ Normative futility ∆ Value futility ∆ Value dependent
Points to the probability of producing physiological effects Points to the success rate of a treatment	Points to the value of achieving a certain goal
Requires medical knowledge to decide upon continuation or discontinuation of treatments	Requires knowing patients’ and their family members’ values and beliefs to decide upon continuation or discontinuation of treatments

**Table 4:** Patient-related/disease-related conditions which affect perceptions of medical futility

**Impossibility of survival**			**Low quality survival**
**Brain death**	**Imminent death**	**Lethal condition**	**Low quality of life**
Total brain death (cortex, medulla, and cerebellum). Partial brain death (cortex, medulla, or cerebellum).	The patient will die in the near future (within several hours or days) irrespective of treatments. A terminally-ill patient A dying patient Premature babies with fatal congenital defects (will die within several hours after birth).	The patient is suffering from an underlying condition which will cause a premature death despite receiving treatments A patient with poor prognosis A patient with end-stage disease A patient with metastatic cancer	Patients with stable vegetative state Very old patients suffering from multiple conditions and organ failure Very old patients suffering from advanced dementia Permanent unconsciousness Patient’s dependence on life-sustaining equipment, devices, and medications

Given the ever-changing conditions of patients due to known or unknown causes (43) as well as patients’ unique personal values and preferences, there is no consensus over these factors. According to some authors, prediction of a patient’s death based on disease severity, poor prognosis, and low QOL is not a good criterion for determining futility of treatment procedures (3, 20, 24, 43). Uncertainties of human sciences, unpredictability of the future, the possibility of committing errors while establishing medical diagnoses and determining prognoses (44, 45), and differences in people’s perceptions of optimum QOL can affect judgments about futility of treatments (42).

*Medical goals (therapeutic or palliative)* **:** Medical futility is inherently correlated with the goals of medical treatments. In fact, goals play a central role in defining medical futility, particularly qualitative futility (12). The main problems here are: ‘What is the goal?’ and ‘Who determines the goal and the time for and ways to achieve the goal?’ In other words, the goal and the right to decision-making are the two important criteria for defining and determining medical futility. Therefore, there are many debates between healthcare teams and family members in terms of determining futile treatments and deciding upon continuation or discontinuation of treatments (3, 12, 21). 

Goals can widely range from completely objective (i.e., physiologic) to completely subjective (qualitative and value-dependent). Moreover, they can be either short-term or long-term. Physiological goals can be determined and established solely by physicians. In other words, determining the instances of physiologic and quantitative futility and deciding upon continuation or discontinuation of treatments are among the responsibilities of physicians. For instance, only physicians can decide not to resuscitate a patient with a myocardial rupture. However, qualitative goals need to be established based on patients’ and their family members’ desires and values. In other words, goals may be completely subjective and even in contrast with physicians’ and other healthcare professionals’ values. In this view, determining the instances of futility and deciding upon continuation or discontinuation of treatments are not done solely by physicians, rather patients’ personal values and preferences need to be also taken into account for decision-making (23). For instance, continuing treatments for a patient with end-stage lung cancer may not result in the long-term goals of recovery or hospital discharge. However, it can help the patient and his/her family members achieve their short-term goals such as having an opportunity for being together in the New Year celebration which is going to be held in the next two days (12). Thus, we cannot achieve desirable outcomes if the goal is not established accurately or the means for achieving the goal are not selected carefully. Subsequently, failure to achieve a certain goal may be erroneously interpreted as futility or worthlessness (15). 

*The value system of patients and their family members, and healthcare professionals* **:** The goals and the benefits as well as the value of achieving them are always affected by patients’ and their family members’, and healthcare professionals’ personal, cultural, socioeconomic, and religious values (3, 7, 8, 12, 22, 26, 46-49). Moreover, patients’ conditions, personal preferences, priorities, and values can affect judgments about the futility of a treatment. Given the importance of the benefits of medical treatments to patients, considering patients’ values may result in decisions which are based on unrealistic or even subjective benefits. For instance, the family members of a patient with brain death may ask for the administration of a completely ineffective traditional medication. Despite the known ineffectiveness of the medication, its administration helps the patient’s family members feel that they did all their best in order to save their patient’s life (37). Another patient may ask for an in vitro fertilization despite knowing its ineffectiveness. Similarly, such a request gives her the lifelong feeling that she has not disregarded any endeavor to have a baby (13). Therefore, preferring a benefit over another is an arbitrary value judgment (22).

***The scope of medical futility***

Our literature review revealed that medical futility debates revolve around two main areas including futility in terminal situations and futility in non-terminal situations (50). Although, futility is a major challenge in ICUs and focuses on end-of-life care (6, 22, 30, 40, 44, 46), it is not unique to terminally-ill patients. Rather, many diagnostic and therapeutic procedures which are performed in non-terminal situations may relate in some ways to futility (50). Two instances of futility in non-terminal situations may include prescribing a non-indicated computed tomography scan for a trauma patient whose chest X-ray shows no pulmonary problem or performing a thyroidectomy on a patient whose hyperthyroidism had been successfully managed by medication therapy and had no manifestation of malignancy.

On the other hand, although medical futility can be related to different preventive, diagnostic, therapeutic, and rehabilitative factors (8, 37), our literature review indicated that it mainly deals with life-sustaining treatments (such as cardiopulmonary resuscitation/the use of ventilator) in end-of-life situations (6, 19, 22, 30, 40, 44, 46, 50, 51), particularly, performing cardiopulmonary resuscitation on patients suffering from terminal cancers (10, 12, 18, 52).

***Reasons behind providing futile medical treatments***

The most important reasons behind providing futile medical treatments which had been referred to either implicitly or explicitly in the literature were as follows:

- Patients’/family members’ request and persistence (2, 6-8, 30, 40, 44, 53)
- Healthcare professionals’ personal emotions, beliefs, and attitudes (6-8, 30, 40, 53)
- Organizational factors and fear over getting involved in medical litigation (2, 6-8, 30, 40, 44, 53)
- Social, cultural, and religious factors (2, 6-8, 30, 40, 44, 53, 54)

***The consequences of providing futile medical treatments***

The most important consequences of providing futile medical treatments which had been mentioned in the literature either implicitly or explicitly were

- Suffering for the patient (2, 54-56);
- Suffering, moral distress, job burnout, job dissatisfaction, and increased turnover among nurses and physicians, and hence, decreased quality of care (2, 9, 11, 40, 49, 54-58);
- Heavy financial burdens on families, healthcare systems, and societies (2, 8, 11, 22, 55, 56, 59);
- Putting other patients at risk (5, 8, 54, 55).

***Challenges related to medical futility***

*The overlap of medical futility and rationing* **:** When expensive diagnostic or therapeutic procedures are prescribed for patients, particularly in ICUs, the two concepts of futility and rationing are usually mistaken for each other. Accordingly, differentiating these two concepts seems essential. In medical futility, prescribing a certain procedure for a certain patient is useless irrespective of the costs of that procedure or the necessity for fair distribution of resources. On the other hand, in rationing, the procedure would be useful to that certain patient; however, it is neither appropriate nor reasonable to implement the procedure for that patient once its costs or other patients’ need for that procedure are taken into account (17, 60). The important point here is that futile treatments should be avoided not because they are expensive, but because they are not useful to the intended patient and are not effective in achieving the intended goals. Moreover, treatments which are useful, but are expensive should also be avoided occasionally because their benefits are not proportionate to their costs (21). Another difference between futility and rationing is that decisions about futility are made at the bedside of a specific patient while rationing-related decisions are made at a community level, based on the needs of different patient populations, and in order to ensure fair distribution of resources in the community. It is noteworthy that futility-related policies should not be considered as a means for managing costs (17, 21, 60), because one of the most important concerns in the area of futility is that some treatments may be labeled as futile in order to cut healthcare costs (14). 

*Lack of objective and valid criteria for determining futility* **:** There is no laboratory test or clinical criteria for accurately identifying patients receiving futile treatments (44). In addition, due to the subjectivity (19, 49, 61), complexity (8, 40, 61, 62), and ambiguity of the concept of medical futility, it is perceived and defined differently by individuals (2, 44, 49, 61, 63). Consequently, assessing the concept solely from the perspectives of healthcare professionals would not be valuable, because their perspectives toward utility and outcome may be different from that of patients and their family members. The type, the amount of the benefit, and the outcomes of medical treatments should be assessed based on the values, preferences, priorities, and desires of patients and family members (2, 26, 40). A major ethical dilemma is: ‘Who has the competence to determine the usefulness and the fruitfulness of treatments and care services?’ This dilemma has remained unresolved because personal, cultural, and religious values and beliefs as well as socioeconomic factors severely affect its perception and interpretation (3, 7, 8, 22, 26, 46-48). 

*The failure of the ICU scoring system to determine the instances of futility* **:** Some researchers introduced poor prognosis, minimal survival chance, and high probability of death as the predictors for futility and recommended the ICU scoring system for determining instances of futility (64). In other words, they attempted to correlate the scores of the ICU scoring system with the instances of futility in order to have permission for withholding and withdrawing of treatments in ICUs. Instruments such as the ICU scoring system are usually used for assessing patients during the first 24 hours after ICU admission, determining the severity of their conditions, determining the type of treatments needed, determining prognosis for patients, and estimating the probability of death based on a series of physiological parameters. However, some other researchers believe that, as these instruments are based solely on physiological parameters, they cannot be used for determining the futility of diagnostic and therapeutic procedures (24). Therefore, using these instruments for determining futility was criticized severely, because the studies showed that

- First, models and systems which determine the severity of illnesses are instruments for estimating hospital death among critically-ill patients. Moreover, their validity has been evaluated in large samples and in certain confidence intervals. Consequently, on an individual level, they should be used cautiously. Once the concepts of probability and confidence interval are accurately explained by physicians and understood by patients and family members, the data obtained from such scoring systems can provide only useful, but not authoritative, information for deciding upon continuation or discontinuation of treatments. The reason is that survival rate (which is determined by these instruments) is only one of the factors in the determination of the appropriateness of treatments for a patient in the ICU. Moreover, these instruments cannot provide information about other factors which are important to clinical decision-making (such as patients’ post-ICU conditions as well as their and their family members’ preferences and goals) (24, 44). Studies showed that patients’ and their family members’ evaluation of treatment options vary with progressive deterioration of patient’s health. In other words, compared with healthy people (such as physicians and nurses), a patient with a critical illness is more likely to choose sophisticated treatments which have low potential benefits. For patients and their family members, a chance of one percent is much better than no chance, and hence, their viewpoints need to be taken into account by healthcare professional while deciding upon the futility or non-futility of treatments (3, 23).
- Second, disease severity, poor prognosis, and probability of death cannot be strong and valid predictors of futility (3, 20, 24, 43). Continuous alterations in patients’ conditions due to either known or unknown causes (43) as well as the inability of illness severity scoring models and systems to provide information about post-ICU morbidity are among the limitations of such instruments in determining the instances of futility. Therefore, decisions upon discontinuing treatments based on the findings of these instruments would be unwise and questionable. On the other hand, the concept of futility is based on value judgments made by different parties, such as patients, family members, and healthcare professionals (2, 22, 23). Hence, it cannot be determined and directly measured based solely on physiological parameters. To conclude, although these systems are helpful for deciding upon the most effective treatments, they cannot be used independently for determining futility and making decisions about continuation or discontinuation (or withholding and withdrawing) of treatments in ICUs (24).
- Third, any attempt to determine futile treatments is associated with the possibility of self-fulfilling prophecy. This problem can affect any situation in which there is a high probability of death and can result in decisions about restricting life-sustaining medical treatments. The risk of self-fulfilling prophecy is that restricting life-sustaining treatments due to a high probability of death abnormally increases mortality rate (3). In other words, the information obtained from ICU scoring systems which show a high severity of illness and a high probability of death can enhance the possibility of healthcare professionals’ self-fulfilling prophecy. Once the death of a patient is highly probable, she/he would receive limited intensive care services, and hence, would have greater probability of death (43, 49). According to Wilkinson and Savulescu (2011), self-fulfilling prophecy is associated with higher mortality rate among patients suffering from hemorrhagic stroke and hypoxic brain injuries, critically-ill patients, and even patients with brain death (3).

## Conclusion

Medical futility is an extremely complex, ambiguous, subjective, situation-specific, value-laden, and goal-dependent concept which is almost always surrounded by some degrees of uncertainty. Thus, there is no objective and valid criteria for determination of medical futility. Determining the futility of a certain treatment for a certain patient and deciding upon its continuation or discontinuation have always been difficult and challenging. This concept is affected by many different factors such as physicians’ and patients’ value systems, medical goals, sociocultural and religious context, and individuals’ emotions and personal characteristics. Such characteristics have made it difficult to achieve a clear consensus over the concept of medical futility. Accordingly, medical futility should be defined and determined at individual level and based on each unique case. The most important reasons behind providing futile medical treatments are patients’/family members’ request and persistence, healthcare professionals’ personal motives, and social, cultural, religious, and organizational factors predominating the immediate community. On the other hand, the most important deleterious consequences of providing futile treatments are suffering for patients, and heavy financial burdens on families, healthcare systems, and societies, and moral distress, job burnout, job dissatisfaction, and increased turnover among healthcare professionals, and hence, decreased care quality. It is essential to study the nature and the mechanism of futile medical treatments in the sociocultural context of each community. The findings of this study can enhance healthcare professionals’ understanding and knowledge of the nature, definitions, attributes, reasons, and consequences of the concept of medical futility. 
